# Prediction of the Surface Roughness in Ultrasonic Vibration-Assisted Grinding of Dental Zirconia Ceramics Based on a Single-Diamond Grit Model

**DOI:** 10.3390/mi12050543

**Published:** 2021-05-10

**Authors:** Xingzhi Xiao, Gang Li, Zhihua Li

**Affiliations:** School of Mechanical Engineering, Nanjing University of Science and Technology, Nanjing 210094, China; jingganglee@outlook.com (G.L.); lzhnjust@126.com (Z.L.)

**Keywords:** comprehensive model, dental ceramics, single-diamond grit, surface roughness, ultrasonic vibration-assisted grinding

## Abstract

Ultrasonic vibration-assisted grinding (UVAG) is regarded as a superior method for the fabrication of ceramic dentures, due to its outstanding performance in hard and brittle materials’ machining. The surface roughness of dentures has a critical effect on the bonding and wear performance between dentures and natural teeth. Accomplishing the prediction of surface roughness will promote the application of UVAG in dental restoration significantly. However, the investigation about surface roughness modeling in the UVAG of ceramics is limited. In this study, a comprehensive surface roughness model was proposed with the consideration of the diamond grits’ random distribution, brittle fracture removal, and ultrasonic vibration characteristics. Based on the indentation fracture removal mechanism, the material removal process was modeled. Rayleigh’s probability density function was introduced to characterize the random distribution of the grits. Besides, the ultrasonic vibration was considered via the analysis of the single-diamond grit motion. Finally, the comprehensive model was developed with the consideration of all the diamond grits. Afterward, the verification experiments were carried out. The experimental results agreed well with the model predictions. Therefore, the comprehensive model can be applied to evaluate the surface roughness and can provide an in-depth understanding of the surface formation in the UVAG of ceramics.

## 1. Introduction

Zirconia ceramics have been widely used in prosthodontics due to their superior biocompatibility, outstanding aesthetics, sufficient mechanical strength, and excellent wear resistance [1]. Meanwhile, because of their high hardness and low fracture toughness, ultrasonic vibration-assisted grinding (UVAG) technology has been introduced to fulfill the direct machining of dental ceramics [2]. As a hybrid machining method, UVAG has been proven to be an effective processing technology for hard and brittle materials [3,4]. The surface roughness of dentures (ceramic crowns, inlays, and implants) has a vital effect on oral health, wear performance, and interfacial bonding properties between dentures and natural teeth, which affects the service performance eventually [5]. Therefore, further studies, in particular for the modeling and prediction of surface roughness in the UVAG of dental ceramics, should be carried out to improve the service life of dentures.

Extensive experimental studies have been conducted on surface roughness in the UVAG of brittle materials, and the influences of machining variables (spindle rotational speed, feed rate, and cutting depth) on surface roughness have been revealed [6,7,8]. With the assistance of ultrasonic vibration, the surface quality is better compared to conventional grinding [9]. For dental ceramics, the machined surface roughness has a crucial effect on the service life of ceramic dentures. The outer surface roughness of the dentures plays an important role in friction and wear performances between the dentures and the natural teeth, while the inner surface roughness of the dentures determines the adhesive properties between the dentures and the substrates [10]. Hence, realizing the prediction of surface roughness will be beneficial to obtain superior wear performances and adhesive properties. However, the current investigations for the surface roughness prediction in the UVAG of brittle materials are limited. Chen et al. [11] proposed a mathematical simulation model to predict the surface roughness in UVAG by dividing up the workpiece into a grid and calculating the minimum value of all diamond grits left at each grid point. The model was based on the plastic shear removal mechanism, which is inappropriate for brittle materials’ machining. Zhang et al. [12] proposed a statistical predictive model based on the random distribution of diamond grits in the UVAG of silica glass. It was assumed that the material removed coincided with the overlapping volume between diamond grits and the workpiece, which is inconsistent with the actual material removal mode. Although several surface roughness models for traditional grinding of ceramics have been developed, the ultrasonic vibration characteristics were not included [13,14].

Diamond grits’ random distribution, brittle fracture removal, and ultrasonic vibration are the three typical and important characteristics in the UVAG of brittle materials. To reveal the surface roughness formation theoretically and predict the surface roughness accurately, the effects of these three characteristics should be considered during modeling. However, the existing research in the UVAG of brittle materials did not consider all three factors at the same time. Therefore, a comprehensive model is urgently required to reveal the formation mechanism and fulfill the effective prediction of surface roughness in the UVAG of brittle materials. More specifically, the surface roughness discussed in this paper is the arithmetic mean deviation of the assessed profile ($R_{a}$). Based on the probabilistic approach of surface roughness prediction used in conventional grinding [15], a comprehensive model was proposed with the integration of the mentioned three factors. The grits random distribution was characterized by the probability density function of the chip thickness. The generation and propagation of the lateral cracks were modeled to clarify the material brittle fracture removal mechanism. Besides, with the kinematic analysis of a single-diamond grit, the ultrasonic vibration characteristics were considered. Finally, the prediction model was developed, and then, the pilot experiments were conducted to verify the model.

The paper is organized into five sections. Following this Introduction section, Section 2 models the material brittle fracture removal process. The comprehensive model for the surface roughness prediction is developed step-by-step in Section 3. In Section 4, pilot experiments are conducted to verify the developed model. Conclusions are drawn in Section 5.

## 2. Modeling of the Material Brittle Fracture Removal Process

Surface roughness is the representation of surface quality, which is decided by the surface formation process. Therefore, the material removal mechanism should be clarified prior to the modeling. Zirconia ceramics, as one of the typical brittle materials, have different material removal mechanisms compared with metals. According to the existing studies, two different material removal modes are discovered in ceramic machining. They are ductile removal and brittle fracture removal, respectively, which are determined by the undeformed chip thickness [16].

**Figure 1:**
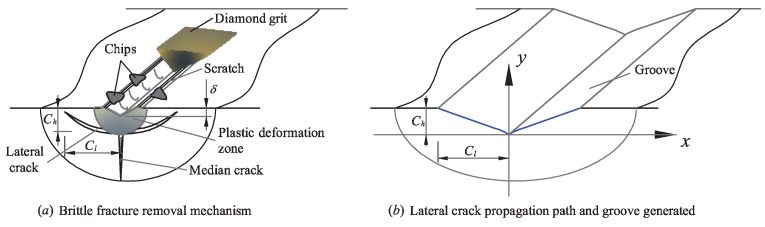
Brittle fracture removal mechanism and the lateral crack propagation path.

**Figure 2:**
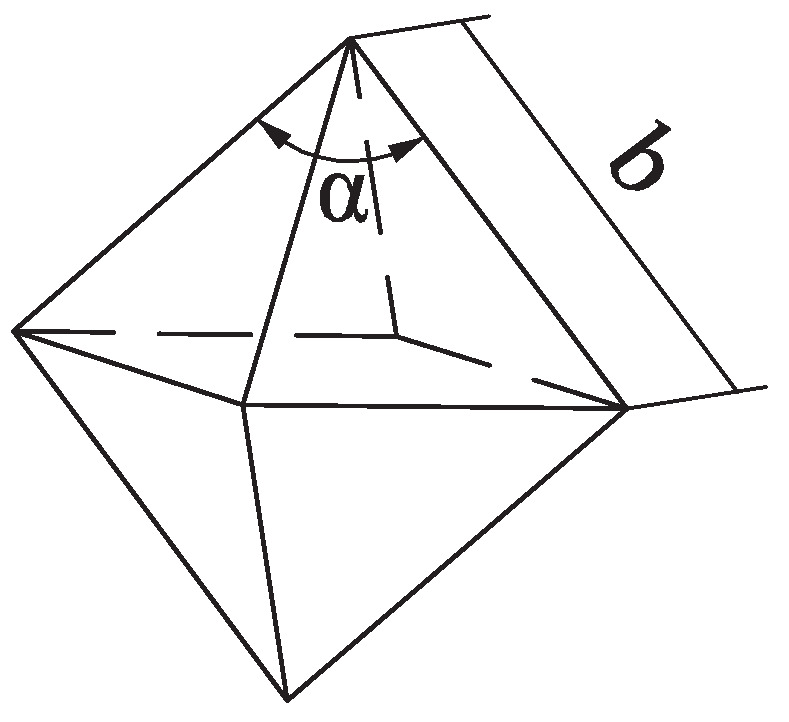
Illustration of the single-diamond grit.

In this paper, only the dominating brittle fracture removal mode was considered. This method has been used and validated in other studies [17,18]. The brittle fracture removal in the grinding of ceramics is likened to the indentation fracture process, as illustrated in Figure 1a. As the diamond grit cuts the workpiece gradually, the deformation zone is formed under the diamond grit. Then, the medial crack is generated and propagates toward the inner part of the workpiece. The lateral cracks initiate and propagate as the diamond grit leaves the workpiece gradually. Finally, the material removal is formed as the lateral cracks reach the workpiece surface. The length and depth of the lateral cracks can be calculated as Equations (1) and (2) [19]. The diamond grits were assumed to be rigid octahedrons of the same size, as shown in Figure 2.

(1) $$C_{l}=C_{2}{\left(\cot\frac{\alpha }{2}\right)}^{\frac{5}{12}}\cdot {\left[\frac{E^{\frac{3}{4}}}{H_{v}K_{IC}{\left(1-v^{2}\right)}^{\frac{1}{2}}}\right]}^{\frac{1}{2}}\cdot F_{n}^{\frac{5}{8}}$$

(2) $$C_{h}=C_{2}{\left(\cot\frac{\alpha }{2}\right)}^{\frac{1}{3}}\cdot \frac{E^{\frac{1}{2}}}{H_{v}}\cdot F_{n}^{\frac{1}{2}}$$

where $H_{v}$ is the hardness of the workpiece material in MPa; $K_{IC}$ is the fracture toughness of the workpiece material in MPa·m${}^{1/2}$; α is the angle between two opposite edges of a diamond grit in (α=π/2 rad); *E* is the Young’s modulus of the workpiece material in MPa; *v* is the Poisson’s ratio of the workpiece material; $F_{n}$ is the load applied to the abrasive grit in N; $C_{2}$ is a dimensionless constant, which is independent of the material-indenter system, and $C_{2}=0.226$ [20].

The relationship between the load applied to the single-diamond grit $F_{n}$ and the groove depth δ can be described as [20]:

(3) $$F_{n}=\frac{1}{2}\xi \delta^{2}\tan^{2}\frac{\alpha }{2}H_{v}$$

where ξ is the geometric factor of the indenter [19]; δ is the penetration depth of the diamond grit into the workpiece in mm. Substituting Equation (3) into Equations (1) and (2), $C_{l}$ and $C_{h}$ can be expressed as:

(4) $$\begin{array}{ccc} C_{l} & = & C_{2}{\left(\cot\frac{\alpha }{2}\right)}^{\frac{5}{12}}\cdot {\left[\frac{E^{\frac{3}{4}}}{H_{v}K_{IC}{\left(1-v^{2}\right)}^{\frac{1}{2}}}\right]}^{\frac{1}{2}}\cdot {\left(\frac{1}{2}\xi \delta^{2}\tan^{2}\frac{\alpha }{2}H_{v}\right)}^{\frac{5}{8}}\\ & = & {\left(\frac{1}{2}\right)}^{\frac{5}{8}}C_{2}{\left(\tan\frac{\alpha }{2}\right)}^{\frac{5}{6}}\xi^{\frac{5}{8}}\frac{E^{\frac{3}{8}}H_{v}^{\frac{1}{8}}}{K_{IC}^{\frac{1}{2}}{\left(1-v^{2}\right)}^{\frac{1}{4}}}\cdot \delta^{\frac{5}{4}}\\ & = & C_{3}\cdot \delta^{\frac{5}{4}} \end{array}$$

(5) $$\begin{array}{ccc} C_{h} & = & C_{2}{\left(\cot\frac{\alpha }{2}\right)}^{\frac{1}{3}}\cdot \frac{E^{\frac{1}{2}}}{H_{v}}\cdot {\left(\frac{1}{2}\xi \delta^{2}\tan^{2}\frac{\alpha }{2}H_{v}\right)}^{\frac{1}{2}}\\ & = & {\left(\frac{1}{2}\right)}^{\frac{1}{2}}C_{2}{\left(\tan\frac{\alpha }{2}\right)}^{\frac{2}{3}}\xi^{\frac{1}{2}}\frac{E^{\frac{1}{2}}}{H_{v}^{\frac{1}{2}}}\cdot \delta \\ & = & C_{4}\cdot \delta \end{array}$$

where $C_{3}={\left(\frac{1}{2}\right)}^{\frac{5}{8}}C_{2}{\left(\tan\frac{\alpha }{2}\right)}^{\frac{5}{6}}\xi^{\frac{5}{8}}\frac{E^{\frac{3}{8}}H_{v}^{\frac{1}{8}}}{K_{IC}^{\frac{1}{2}}{\left(1-v^{2}\right)}^{\frac{1}{4}}}$ and $C_{4}={\left(\frac{1}{2}\right)}^{\frac{1}{2}}C_{2}{\left(\tan\frac{\alpha }{2}\right)}^{\frac{2}{3}}\xi^{\frac{1}{2}}\frac{E^{\frac{1}{2}}}{H_{v}^{\frac{1}{2}}}$. The initiation and propagation of the lateral cracks result in the material removal when the cracks reach the workpiece surface. According to the expressions of the crack length $C_{l}$ and the crack depth $C_{h}$, the mathematical relationship between $C_{l}$ and $C_{h}$ can be derived. This means that the crack propagation path can be expressed as a formula. Assuming that $y=C_{h}=C_{4}\cdot \delta ,x=C_{l}=C_{3}\cdot \delta^{\frac{5}{4}}$, the penetration depth of the diamond grit can be easily expressed in the following two ways:

(6) $$\delta =\frac{y}{C_{4}}$$

(7) $$\delta ={\left(\frac{x}{C_{3}}\right)}^{\frac{4}{5}}$$

Equating Equation (6) to Equation (7), the expression of the crack propagation path can be obtained as:

(8) $$y=\frac{C_{4}}{C_{3}^{\frac{4}{5}}}\cdot x^{\frac{4}{5}}$$

Substituting the expressions of $C_{3}$ and $C_{4}$ into Equation (8) and also considering that the value of *y* should be positive, the final function can be expressed as:

(9) $$\begin{array}{ccc} y & = & C_{2}^{\frac{1}{5}}\frac{E^{\frac{1}{5}}K_{IC}^{\frac{2}{5}}{\left(1-v^{2}\right)}^{\frac{1}{5}}}{H_{v}^{\frac{2}{5}}}\cdot x^{\frac{4}{5}}\\ & = & a\cdot x^{\frac{4}{5}} \end{array}$$

where $a=C_{2}^{\frac{1}{5}}\frac{E^{\frac{1}{5}}K_{IC}^{\frac{2}{5}}{\left(1-v^{2}\right)}^{\frac{1}{5}}}{H_{v}^{\frac{3}{5}}}$. The specific expression of the lateral crack propagation path was proposed based on the brittle fracture removal mechanism, and the shape of the crack propagation path is shown in Figure 1b. This equation was utilized as the foundation to develop the prediction model of the surface roughness in the UVAG of zirconia ceramics.

## 3. Development of the Prediction Model of the Surface Roughness in the UVAG of Zirconia Ceramics

UVAG might be considered as a combination of ultrasonic machining and conventional grinding [21]. The final surface formed in the UVAG of ceramics is decided by the combined cutting of numerous grits. Due to the random distribution of grits in the axial and radial directions, the penetration depth of each grit is different, and overlapping appears among the grooves. Meanwhile, the tool motion also causes the grooves to overlap. The degree of overlapping affects the final surface formation directly, so it is crucial to combine these effects in the final prediction model of the surface roughness. To get a realizable and reliable prediction model of the surface roughness, some assumptions and simplifications are needed:

1. The diamond grits were assumed to be rigid octahedrons of the same size, as shown in Figure 2. Every four adjacent triangles had a common vertex, forming a pyramid. Only one pyramid of each octahedral particle took part in cutting; the other was buried in the metal bond.
2. The edge lengths of the single-diamond grit were assumed to be the same *b*.
3. For each pair of adjacent grits, the grooves generated by them only had one overlap, and the overlapping degrees for every adjacent groove were the same.

The definition of the surface roughness $R_{a}$ is presented initially in Section 3.1. Then, the random distribution of the diamond grits is characterized in Section 3.2. Section 3.3 describes the derivation process of the expected value of the surface roughness $E(R_{a})$. The parameter σ, which defines the probability density function, is calculated in Section 3.4. Finally, the comprehensive prediction model of the surface roughness is proposed in Section 3.5 with the consideration of the tool motion effect and tool wear.

### 3.1. Definition of the Surface Roughness $R_{a}$

Surface roughness is an index to characterize the surface quality quantitatively, and it is normally defined as [22]:

(10) $$R_{a}=\frac{1}{l}\int_{0}^{l}\left|y-y_{cl}\right|dl$$

where *l* is the evaluation length; $y_{cl}$ is the position of the center line so that the areas above and below the line are equal.

The statistical expression of $R_{a}$ can be described as [22]:

(11) $$R_{a}=\frac{1}{l}\int_{y_{min}}^{y_{max}}\left|y-y_{cl}\right|p\left(y\right)dy$$

where $y_{max}$ and $y_{min}$ are the highest and lowest peak height of the surface profile; p(y) is the probability to get a peak of height *y*.

### 3.2. Grits’ Random Distribution on the Tool Used in the UVAG of Ceramics

**Figure 3:**
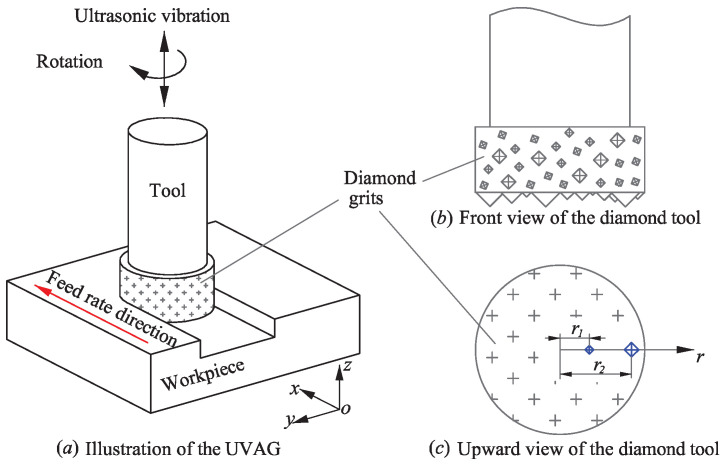
Random distribution of the grits on the UVAG tool.

**Figure 4:**
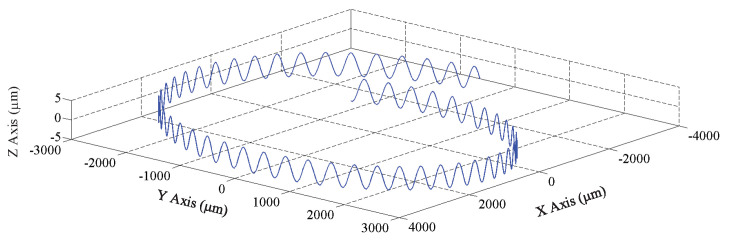
Trajectory of a diamond grit in UVAG.

For grinding tools used in the UVAG of ceramics, the diamond grits are sintered randomly on the lateral and end faces of the metal shank, as illustrated in Figure 3a,b. The trajectory of a diamond grit in UVAG is shown in Figure 4. The motion of the diamond grit consists of the spindle rotation, spindle ultrasonic vibration, and horizontal feed motion of the tool. During the UVAG of zirconia ceramics, the diamond grits on the end face take part in cutting, and the random distribution in the axial direction results in different penetration depths for each grit. This means that the undeformed chip thickness or penetration depth of the grits is not fixed, although the cutting depth of the tool is set as constant. This is a typical characteristic in the UVAG of ceramics, and it should be considered in the comprehensive surface roughness model. Thereby, Rayleigh’s probability density function proposed by Younis and Alaw [23] was introduced to describe the penetration depth δ as follows:

(12) $$f\left(\delta \right)=\left\{\begin{array}{c} \left(\frac{\delta }{\sigma^{2}}\right)e^{-\frac{\delta^{2}}{2\sigma^{2}}}\;\delta \geq 0\\ 0\;\;\;\delta <0 \end{array}\right.$$

where σ is a parameter that defines the probability density function completely and depends on the machining conditions.

The expected value and the standard deviation of the penetration depth δ can be expressed as:

(13) $$E\left(\delta \right)=\sqrt{\frac{\pi }{2}}\cdot \delta$$

(14) $$sd\left(\delta \right)=\sqrt{\frac{4-\pi }{2}}\cdot \delta$$

Besides the random distribution in the axial direction, the diamond grits are also distributed irregularly in the radial direction. This leads to the overlapping of grooves in the radial direction, which affects the surface formation subsequently. As shown in Figure 3c, from the bottom view of the ultrasonic tool, the diamond grits are randomly positioned on the end face of the tool, while they can be generally considered as uniformly distributed on the tool end face. In that case, the diamond grits are also distributed uniformly in the radial direction of the end face. Coordinate *r* is the position of the grits in the radial direction, so the probability density function of *r* is given by:

(15) $$f\left(r\right)=\frac{1}{R}\;\;\;0<r<R$$

where *R* is the radius of the tool in mm.

**Figure 5:**
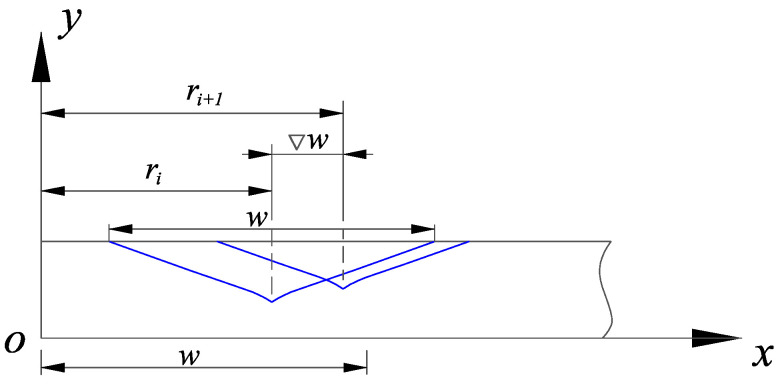
Section view of two adjacent grooves.

The groove overlapping is mainly caused by the grit random distribution and the tool motion. In this subsection, the effect of the grit random distribution was analyzed first. From the section view of the grooves in Figure 5, two successive grooves produced by adjacent grits are illustrated. The center distance ∇w of these two grooves is introduced to characterize the overlapping.

(16) $$\nabla w=\left|r_{i+1}-r_{i}\right|$$

where *i* denotes the *i*-th groove.

Let $w_{1}=r_{i+1}-r_{i}$ and $w_{2}=r_{i}$, so the expressions of $r_{i}$ and $r_{i+1}$ can be written as:

(17) $$r_{i}=w_{2}\;and\;r_{i+1}=w_{1}+w_{2}$$

To calculate the expected value of ∇w, a joint probability density function is required, which can be expressed as [15]:

(18) $$f\left(w_{1},w_{2}\right)=f\left(r_{1}\left(w_{1},w_{2}\right),r_{2}\left(w_{1},w_{2}\right)\right)\left|J\right|$$

where *J* is the Jacobian determinant [15].

Based on Equation (15), the probability density function of $w_{1}$ for the section length *w* is defined by the following two equations:

(19) $$f\left(w_{1}\right)=\int_{w_{1}}^{w}f\left(w_{1},w_{2}\right)dw_{2}\;\;\;w_{1}>0$$

(20) $$f^{{}^{\prime }}\left(w_{1}\right)=\int_{-w_{1}}^{w}f\left(w_{1},w_{2}\right)dw_{2}\;\;\;w_{1}<0$$

As defined in Equation (16), the center-to-center distance of two adjacent grooves ∇w is equal to $w_{1}$. Therefore, the probability density function of the distance ∇w is equal to the combination of the probability density function of $w_{1}$ in positive and negative conditions, which can be written as:

(21) $$f\left(\nabla w\right)=f\left(w_{1}\right)+f^{{}^{\prime }}\left(w_{1}\right)$$

According to the above probability density function, the expected value of the center-to-center distance for successive grooves in the radial direction E(∇w) can be derived as:

(22) $$E\left(\nabla w\right)=\int_{0}^{w}\nabla w\cdot f\left(\nabla w\right)d\nabla w=\frac{1}{3}w$$

**Figure 6:**
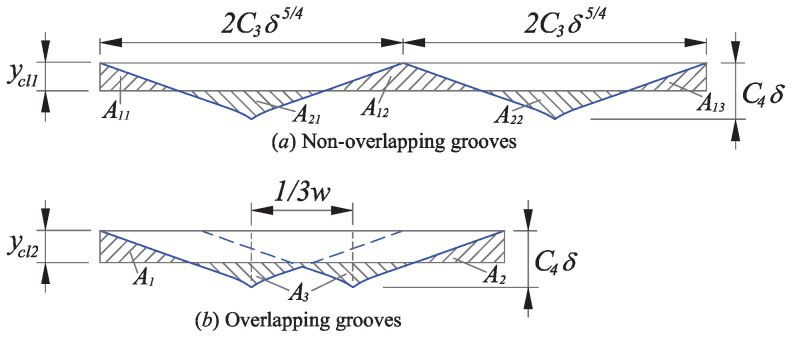
Illustration of non-overlapping and overlapping for two adjacent grooves.

The view of non-overlapping and overlapping grooves can be illustrated in Figure 6a,b, respectively. The center lines of non-overlapping and overlapping grooves $y_{cl1}$ and $y_{cl2}$ can be calculated according to the definition of the center line described in Equation (10). Then, the overlapping factor can be derived with the comparison of the non-overlapping and overlapping groove sections.

According to the definition of $y_{cl1}$, the areas above and below the center line are equal, which can be expressed as:

(23) $$A_{11}+A_{12}+A_{13}=A_{21}+A_{22}$$

specified as:

(24) $$\begin{array}{c} 4\left(C_{3}y_{cl1}\cdot \delta^{\frac{5}{4}}-\left(C_{5}\cdot \delta^{\frac{9}{4}}-\left(\left(C_{4}\delta -y_{cl1}\right){\left(\frac{C_{4}\delta -y_{cl1}}{a}\right)}^{\frac{5}{4}}-\frac{5}{9}a{\left(\frac{C_{4}\delta -y_{cl1}}{a}\right)}^{\frac{9}{4}}\right)\right)\right)\\ =2\left(C_{3}y_{cl1}\cdot \delta^{\frac{5}{4}}-\left(C_{3}C_{4}-\frac{5}{9}aC_{3}^{\frac{9}{5}}\right)\cdot \delta^{\frac{9}{4}}\right) \end{array}$$

and simplified as:

(25) $$y_{cl1}=\frac{C_{5}}{C_{3}}\cdot \delta$$

where the areas $A_{11}$, $A_{12}$,$A_{13}$, $A_{21}$, and $A_{22}$ are illustrated in Figure 6a and $C_{5}=C_{3}C_{4}-\frac{5}{9}aC_{3}^{9/5}$.

A similar method is used to get $y_{cl2}$.

(26) $$y_{cl2}=C_{4}\delta -\frac{5}{12}\left[{\left(\frac{1}{3}\right)}^{\frac{9}{5}}+1\right]aC_{3}^{\frac{4}{5}}\delta$$

As shown in Figure 6, the overlapping degree of the areas above and below the center line was assumed to be constant. In this case, the area above the center line was chosen to calculate the overlapping factor $k_{1}$, which is the ratio of the area with overlapping to the area without overlapping. The specific expression of $k_{1}$ is described as:

(27) $$\begin{array}{ccc} k_{1} & = & \frac{A_{1}+A_{2}}{A_{11}+A_{12}+A_{13}}\\ & = & \frac{C_{3}y_{cl2}\cdot \delta^{\frac{5}{4}}-C_{5}\cdot \delta^{\frac{9}{4}}+\frac{1}{a^{\frac{5}{4}}}{\left(C_{4}\delta -y_{cl2}\right)}^{\frac{9}{4}}-\frac{5}{9a^{\frac{5}{4}}}{\left(C_{4}\delta -y_{cl2}\right)}^{\frac{9}{4}}}{2\left(C_{3}y_{cl1}\cdot \delta^{\frac{5}{4}}-C_{5}\cdot \delta^{\frac{9}{4}}+\frac{1}{a^{\frac{5}{4}}}{\left(C_{4}\delta -y_{cl1}\right)}^{\frac{9}{4}}-\frac{5}{9a^{\frac{5}{4}}}{\left(C_{4}\delta -y_{cl1}\right)}^{\frac{9}{4}}\right)} \end{array}$$

Substituting Equations (25) and (26) into Equation (27), the value of $k_{1}$ can be obtained:

(28) $$k_{1}=0.5914$$

### 3.3. Expected Value of the Surface Roughness $E(R_{a})$

**Figure 7:**
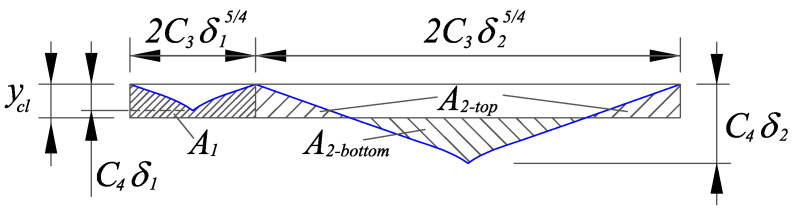
Profile of the grooves generated in the UVAG of ceramics.

As shown in Figure 7, two types of grooves are generated during the UVAG of ceramics, which depend on the relative position between the depth of the radial crack $C_{h}$ and the center line $y_{cl}$. In this section, the center line $y_{cl}$ was calculated to deduce the expected value of the surface roughness $E(R_{a})$.

(29) $$p_{1}E\left(A_{1}\right)+p_{2}E\left(A_{2-top}\right)=p_{2}E\left(A_{2-bottom}\right)$$

where $p_{1}$ and $p_{2}$ are the probabilities of a groove depth to be below or above the center line, respectively.

The specific expression of $p_{1}$ and $p_{2}$ can be derived from the probability density function of the penetration depth f(δ), which can be expressed as:

(30) $$p_{1}=\int_{0}^{y_{cl}}f\left(\delta \right)d\delta$$

(31) $$p_{2}=\int_{y_{cl}}^{\infty }f\left(\delta \right)d\delta$$

Considering the overlapping, the expected value of the area above the center line $E(A_{1})$, in the case that the groove depth $\delta_{1}$ is less than $y_{cl}$, can be expressed as:

(32) $$\begin{array}{ccc} E(A_{1}) & = & k_{1}\cdot 2E\left(C_{l-\delta_{1}}\cdot C_{h-\delta_{1}}-\int_{0}^{C_{l-\delta_{1}}}ax^{\frac{4}{5}}\cdot dx\right)\\ & = & k_{1}\cdot 2E\left(C_{3}y_{cl}\cdot \delta_{1}^{\frac{5}{4}}-\left(C_{3}C_{4}-\frac{5}{9}aC_{3}^{\frac{9}{5}}\right)\cdot \delta_{1}^{\frac{9}{4}}\right)\\ & = & k_{1}\cdot 2\left(C_{3}y_{cl}\cdot E\left(\delta_{1}^{\frac{5}{4}}\right)-C_{5}\cdot E\left(\delta_{1}^{\frac{9}{4}}\right)\right) \end{array}$$

In the case of a groove depth $\delta_{1}$ larger than $y_{cl}$, the expected value of the area above the center line $E(A_{2-top})$ and the expected value of the area below the center line $A_{2-bottom}$ can be described respectively as:

(33) $$\begin{array}{ccc} E(A_{2-top}) & = & k_{1}\cdot 2E\left(C_{3}y_{cl}\cdot \delta_{2}^{\frac{5}{4}}-\left(C_{5}\cdot \delta_{2}^{\frac{9}{4}}-\left(\left(C_{4}\delta_{2}-y_{cl}\right){\left(\frac{C_{4}\delta_{2}-y_{cl}}{a}\right)}^{\frac{5}{4}}\right.\right.\right.\\ & & \left.\left.\left.-\frac{5}{9}a{\left(\frac{C_{4}\delta_{2}-y_{cl}}{a}\right)}^{\frac{9}{4}}\right)\right)\right)\\ & = & k_{1}\cdot 2\left(C_{3}y_{cl}\cdot E\left(\delta_{2}^{\frac{5}{4}}\right)-C_{5}\cdot E\left(\delta_{2}^{\frac{9}{4}}\right)+\frac{4}{9}\cdot \frac{C_{4}^{\frac{9}{4}}}{a^{\frac{5}{4}}}\cdot E\left(\delta_{2}^{\frac{9}{4}}\right)\right.\\ & & \left.-\frac{C_{4}^{\frac{5}{4}}}{a^{\frac{5}{4}}}y_{cl}\cdot E\left(\delta_{2}^{\frac{5}{4}}\right)+\frac{5}{8}\frac{C_{4}^{\frac{1}{4}}}{a^{\frac{5}{4}}}y_{cl}^{2}\cdot E\left(\delta_{2}^{\frac{1}{4}}\right)\right) \end{array}$$

(34) $$\begin{array}{ccc} E(A_{2-bottom}) & = & k_{1}\cdot 2E\left(\left(C_{4}\delta_{2}-y_{cl}\right){\left(\frac{C_{4}\delta_{2}-y_{cl}}{a}\right)}^{\frac{5}{4}}-\frac{5}{9}a{\left(\frac{C_{4}\delta_{2}-y_{cl}}{a}\right)}^{\frac{9}{4}}\right)\\ & = & k_{1}\cdot 2\left(\frac{4}{9}\frac{C_{4}^{\frac{9}{4}}}{a^{\frac{5}{4}}}\cdot E\left(\delta_{2}^{\frac{9}{4}}\right)-\frac{C_{4}^{\frac{5}{4}}}{a^{\frac{5}{4}}}y_{cl}\cdot E\left(\delta_{2}^{\frac{5}{4}}\right)+\frac{5}{8}\frac{C_{4}^{\frac{1}{4}}}{a^{\frac{5}{4}}}y_{cl}^{2}\cdot E\left(\delta_{2}^{\frac{1}{4}}\right)\right) \end{array}$$

The calculation of the expected values above requires the definition of the probability density functions for those cases where the chip thickness is below and above the center line. Therefore, two new probability density functions must be defined in each region as:

(35) $$f_{1}\left(\delta \right)=\frac{f(\delta )}{\int_{0}^{y_{cl}}f\left(\delta \right)d\delta }\;\;\;0\leq \delta <y_{cl}$$

(36) $$f_{2}\left(\delta \right)=\frac{f(\delta )}{\int_{y_{cl}}^{\infty }f\left(\delta \right)d\delta }\;\;\;y_{cl}\leq \delta <\infty$$

Substituting Equations (30)–(36) into Equation (29), the expression of the center line $y_{cl}$ can be deduced:

(37) $$C_{3}y_{cl}\left(p_{1}E\left(\delta_{1}^{\frac{5}{4}}\right)+p_{2}E\left(\delta_{2}^{\frac{5}{4}}\right)\right)=C_{5}\left(p_{1}E\left(\delta_{1}^{\frac{9}{4}}\right)+p_{2}E\left(\delta_{2}^{\frac{9}{4}}\right)\right)$$

and simplified as:

(38) $$y_{cl}=\frac{C_{5}}{C_{3}}\cdot E\left(\delta \right)$$

Combining the area above and below the center line, the expected value for the surface roughness can be expressed as:

(39) $$E\left(R_{a}\right)=p_{1}E\left(R_{a1}\right)+p_{2}E\left(R_{a2}\right)$$

where $E(R_{a1})$ and $E(R_{a2})$ are the expected values of the surface roughness for a groove depth δ below and above the center line.

The expected value of the surface roughness contribution of the grooves’ depths below the center line $E(R_{a1})$ can be calculated by:

(40) $$\begin{array}{ccc} E(R_{a1}) & = & E\left(\frac{A_{1}}{2C_{3}\delta_{1}^{\frac{5}{4}}}\right)\\ & = & k_{1}\cdot \left(y_{cl}-\frac{C_{5}}{C_{3}}\cdot E\left(\delta_{1}\right)\right) \end{array}$$

The expected value of the surface roughness contribution of the grooves’ depths above the center line $E(R_{a2})$ can be described by:

(41) $$\begin{array}{ccc} E(R_{a2}) & = & E\left(\frac{A_{2-top}+A_{2-bottom}}{2C_{3}\delta_{2}^{\frac{5}{4}}}\right)\\ & = & k_{1}\cdot \left(y_{cl}-\frac{C_{5}}{C_{3}}\cdot E\left(\delta_{2}\right)+\frac{8}{9}\cdot \frac{C_{4}^{\frac{9}{4}}}{C_{3}a^{\frac{5}{4}}}\cdot E\left(\delta_{2}\right)\right.\\ & & \left.-2\cdot \frac{C_{4}^{\frac{5}{4}}}{C_{3}a^{\frac{5}{4}}}y_{cl}+\frac{5}{4}\cdot \frac{C_{4}^{\frac{1}{4}}}{C_{3}a^{\frac{5}{4}}}y_{cl}^{2}\cdot E\left(\frac{1}{\delta_{2}}\right)\right) \end{array}$$

The probabilities of the lateral crack depth to be below and above the center line are as follows:

(42) $$p_{1}=\int_{0}^{y_{cl}}f\left(\delta \right)d\delta =1-e^{-\frac{y_{cl}^{2}}{2\sigma^{2}}}$$

The probability of the lateral crack depth above the center line is calculated as:

(43) $$p_{2}=\int_{y_{cl}}^{\infty }f\left(\delta \right)d\delta =1-p_{1}=e^{-\frac{y_{cl}^{2}}{2\sigma^{2}}}$$

Substituting Equations (40)–(43) into Equation (39), the expected value of $E(R_{a})$ can be obtained:

(44) $$E\left(R_{a}\right)=0.3635k_{1}\cdot \sigma$$

### 3.4. Calculation of the Parameter σ

Based on Equation (44), the parameter σ needs to be obtained to get the expected value of surface roughness $E(R_{a})$.

**Figure 8:**
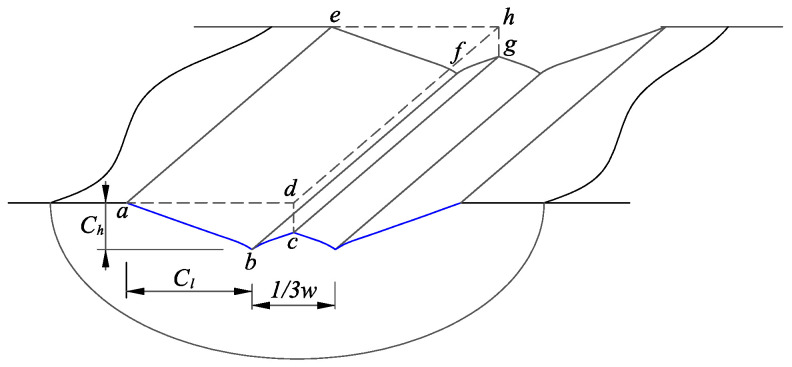
Illustration of the theoretical material removal volume of a single-diamond grit.

With the consideration of the interference between adjacent diamond grits, the schematic illustration of the theoretical volume removed (polyhedron abcd−efgh) can be seen in Figure 8. From the figure, the expected volume removed by a single-diamond grit can be defined as:

(45) $$E\left(V_{s}\right)=\left(C_{5}\cdot E\left(\delta^{\frac{9}{4}}\right)+\frac{1}{3}C_{3}C_{4}\cdot E\left(\delta^{\frac{9}{4}}\right)-\frac{5}{9}{\left(\frac{1}{3}\right)}^{\frac{9}{5}}aC_{3}^{\frac{9}{5}}\cdot E\left(\delta^{\frac{9}{4}}\right)\right)\cdot \frac{2\pi nR}{60}\cdot \frac{E(\delta )}{2Af_{v}}$$

where *n* is the spindle rotational speed in $\min^{-1}$; *A* is the ultrasonic vibration amplitude in µm; $f_{v}$ is the ultrasonic vibration frequency in Hz.

According to the machining parameters, the actual volume removed during UVAG can be obtained as:

(46) $$V_{a}=2R\left(a_{p}+A\right)v_{f}\frac{1}{f_{v}}$$

where $v_{f}$ is the feed rate in mm/min; $a_{p}$ denotes the cutting depth in mm.

The volume removed obtained from theoretical analysis should be equal to the volume removed calculated using the machining parameters. Therefore, equating the theoretical volume removed with combining the diamond grit number to the actual volume removed is described as:

(47) $$\begin{array}{ccc} N_{a}\cdot E\left(V_{s}\right) & = & V_{a}\\ N_{a}\cdot \left(C_{5}+\frac{1}{3}C_{3}C_{4}-\frac{5}{9}{\left(\frac{1}{3}\right)}^{\frac{9}{5}}aC_{3}^{\frac{9}{5}}\right)\cdot E\left(\delta^{\frac{13}{4}}\right)\cdot \frac{2\pi nR}{60}\cdot \frac{1}{2Af_{v}} & = & 2R\left(a_{p}+A\right)v_{f}\frac{1}{f_{v}} \end{array}$$

Thus, the expected value of the penetration depth can be obtained:

(48) $$E\left(\delta^{\frac{13}{4}}\right)=\frac{120\left(a_{p}+A\right)v_{f}A}{\pi n\cdot N_{a}\cdot \left(C_{5}+\frac{1}{3}C_{3}C_{4}-\frac{5}{9}{\left(\frac{1}{3}\right)}^{\frac{9}{5}}aC_{3}^{\frac{9}{5}}\right)}$$

where $E\left(\delta^{\frac{13}{4}}\right)=\int_{0}^{\infty }\frac{\delta^{\frac{17}{4}}}{\sigma^{2}}e^{-\frac{\delta^{2}}{2\sigma^{2}}}d\delta =4.4938\sigma^{\frac{13}{4}}$.

Therefore, σ can be derived from Equation (48) with the following expression:

(49) $$\sigma ={\left(\frac{80\left(a_{p}+A\right)v_{f}A}{3\pi n\cdot N_{a}\cdot \left(C_{5}+\frac{1}{3}C_{3}C_{4}-\frac{5}{9}{\left(\frac{1}{3}\right)}^{\frac{9}{5}}aC_{3}^{\frac{9}{5}}\right)}\right)}^{\frac{4}{13}}$$

### 3.5. Comprehensive Predictive Model for the Surface Roughness

As mentioned above, the tool motion also affects the grooves overlapping and, subsequently, the surface roughness. The tool motion was determined by the cutting parameters (spindle rotational speed *n*, feed rate $v_{f}$, and cutting depth $a_{p}$). Besides, the tool wear was not considered in the above modeling process, which is mainly affected by the machining time *t*. In this case, a parameter $k_{2}=f\left(n,v_{f},a_{p},t\right)$ was introduced to characterize the influence of the tool motion on the grooves overlapping and tool wear. Therefore, the final comprehensive surface roughness model can be described as:

(50) $$E\left(R_{a}\right)=k_{2}\cdot 0.3635k_{1}\sigma$$

Substituting Equations (28) and (49) and the expression of $k_{2}$ into Equation (50), the comprehensive surface model can be rewritten as:

(51) $$\begin{array}{ccc} E(R_{a}) & = & 0.3635\times 0.5914\cdot f\left(n,v_{f},a_{p},t\right)\cdot {\left(\frac{80\left(a_{p}+A\right)v_{f}A}{3\pi n\cdot N_{a}\cdot \left(C_{5}+\frac{1}{3}C_{3}C_{4}-\frac{5}{9}{\left(\frac{1}{3}\right)}^{\frac{9}{5}}aC_{3}^{\frac{9}{5}}\right)}\right)}^{\frac{4}{13}}\\ & = & 0.2150\cdot f\left(n,v_{f},a_{p},t\right)\cdot {\left(\frac{80\left(a_{p}+A\right)v_{f}A}{3\pi n\cdot N_{a}\cdot \left(C_{5}+\frac{1}{3}C_{3}C_{4}-\frac{5}{9}{\left(\frac{1}{3}\right)}^{\frac{9}{5}}aC_{3}^{\frac{9}{5}}\right)}\right)}^{\frac{4}{13}} \end{array}$$

The number of diamond grits $N_{a}$ can be obtained as [15]:

(52) $$\begin{array}{ccc} N_{a} & = & {\left[\frac{0.88\times 10^{-3}}{\frac{\sqrt{2}}{3}b^{3}\rho }\cdot \frac{C_{a}}{100}\right]}^{\frac{2}{3}}\cdot \pi R^{2}\\ & = & C_{0}\cdot \frac{C_{a}^{\frac{2}{3}}}{b^{2}}\cdot \pi R^{2} \end{array}$$

where $C_{a}$ is the diamond grits concentration [17]; ρ is the density of the abrasive material in g/mm${}^{3}$, $\rho =3.25\times 10^{-3}$ g/mm${}^{3}$; $C_{0}$ is a dimensionless constant, $C_{o}={\left[3\times 0.88\times 10^{-3}/\left(100\times 2^{0.5}\rho \right)\right]}^{\frac{2}{3}}=0.033$.

## 4. Experimental Verification

### 4.1. Experimental Setup

**Figure 9:**
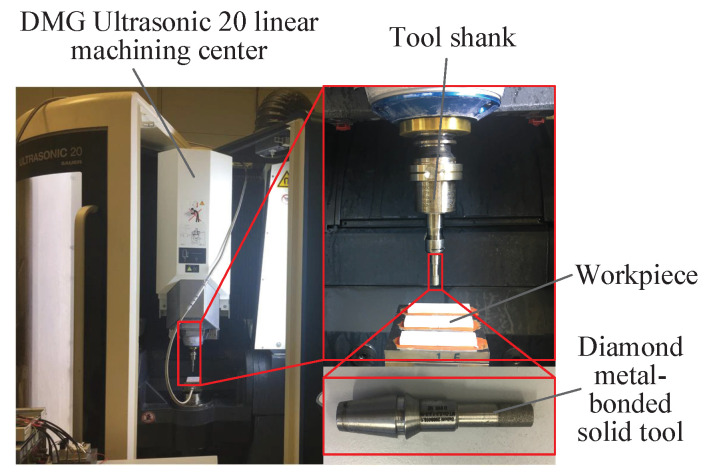
Experimental setup adopted for the UVAG experiments.

As illustrated in Figure 9, the slots were machined with the UVAG method. The machining center (DMG Ultrasonic 20 linear, DMG, Berlin, Germany) mainly consisted of an ultrasonic spindle system, a numerical control machining system, and a coolant system.

**Figure 10:**
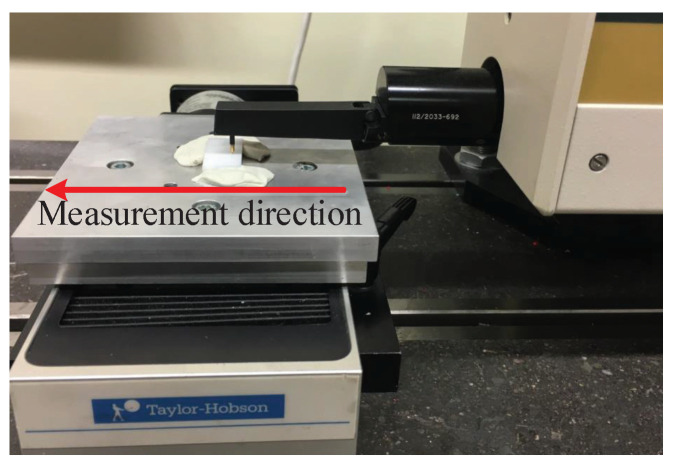
Measurement method adopted after the UVAG experiments.

The maximum spindle rotational speed with ultrasonic vibration was 10,000 $\min^{-1}$, while the maximum spindle rotational speed without ultrasonic vibration was 42,000 $\min^{-1}$. The vibration frequency varied from 20 kHz to 50 kHz for the different tool-workpiece systems adopted. The vibration amplitude was measured by a laser vibrometer (Polytec OFV 353 sensor head and OFV 2200 vibrometer controller). The tool used in the experiments was provided by Schott Diamantwerkzeuge GmbH in Germany, and it was a diamond metal-bonded solid tool with a diameter of 6 mm and a diamond grit size of D91. The Taylor Hobson profilometer was used to measure the surface roughness of the slots, as illustrated in Figure 10. The measurement direction was the same as the feed rate direction. Each slot was measured six times, and the arithmetic average value was obtained as the global surface roughness of the slot. The cut-off was 0.8 mm; the measurement length was 8 mm; and the spacing was 0.8 mm.

### 4.2. Design of Experiments

The workpiece materials were zirconia ceramics provided by Qinhuangdao Aidite High-Technical Ceramics, CO., Ltd (Qinhuangdao, China). The compositions and the primary mechanical properties of the zirconia ceramics are shown in Table 1 and Table 2, respectively. The dimensions of the zirconia ceramics were 30 × 15 × 5 mm.

Twenty-four groups of experiments were carried out. Considering the effect of the tool wear, the odd-group experiments were selected to calibrate $k_{2}$, and all 24 experiments were used to verify the proposed model. The details of the experimental design are shown in Table 3. There were three input variables (*n*, $v_{f}$, and $a_{p}$). Each of these 3 parameters assumed 8 different values, which explained why 24 experiments were performed. Other variables, such as the ultrasonic vibration frequency and ultrasonic vibration amplitude, were kept constant with a value of 25,010 Hz and 5 µm, respectively.

### 4.3. Obtaining $k_{2}$ and Surface Roughness Prediction

**Figure 11:**
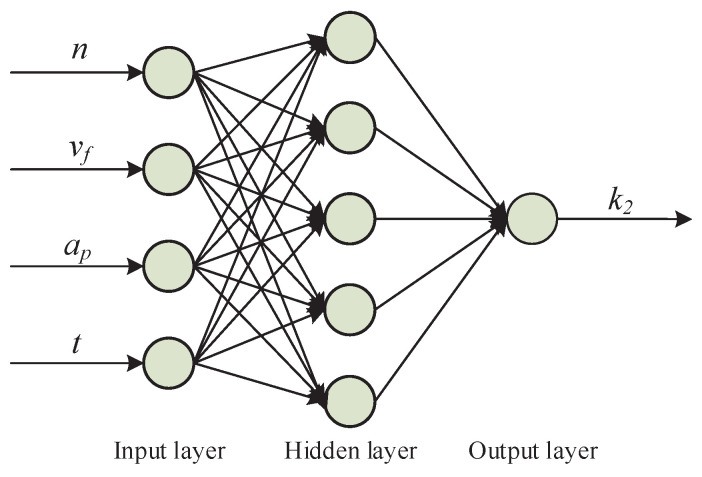
Structure of the implemented backpropagation neural network.

**Figure 12:**
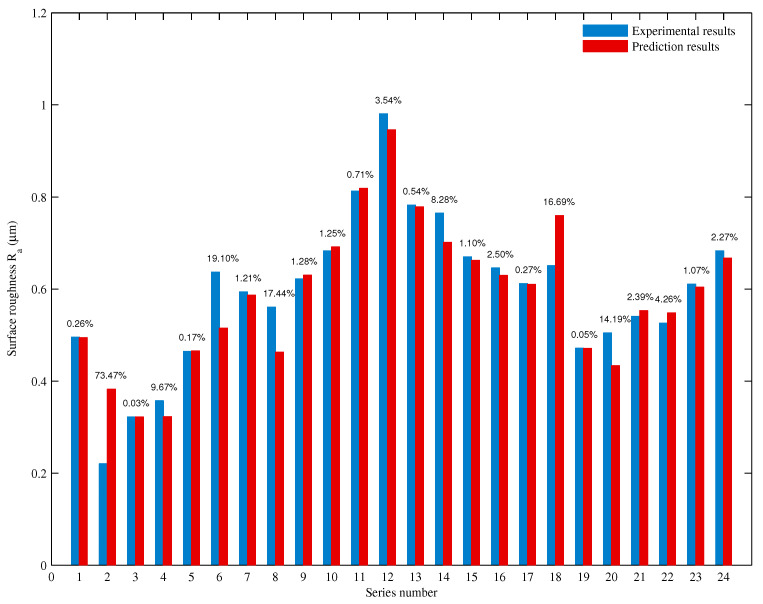
Comparison analysis between prediction and experimental results.

$k_{2}$ contains the effect of the tool motion on the grooves overlapping and tool wear. The tool wear was not linear with time: initially, it increased sharply, then it remained stable, and finally, it rose rapidly again. Therefore, using the linear estimation or least squares estimation to calibrate $k_{2}$ was not effective. In this case, the backpropagation neural network algorithm was chosen to get the function $f(n,v_{f},a_{p},t)$. A typical backpropagation neural network usually consists of an input layer, a hidden layer, and an output layer. According to the expression of $k_{2}$, the number of neurons of the input layer was selected as 4. The number of hidden layers was 1, and the number of neurons was 5. The number of output neurons was 1, and the structure of the backpropagation neural network is shown in Figure 11. Before inputting the training sample, the experimental data needed to be normalized, and the data of each parameter was normalized to the interval (0, 1). The training program was written in MATLAB, and the momentum gradient descent method was used to train the established neural network model. The minimum error of the training target was 0.0005, and the maximum allowed training step size was 200,000 steps. The training model was used to train 12 sets of odd-group experimental data. When the training error was less than the minimum error of the training target, the training ended, and a backpropagation neural network prediction model for $k_{2}$ was formed. Then, the comprehensive predictive model for the surface roughness could be obtained. The comparison between the experimental results and the prediction results is shown in Figure 12.

As shown in Figure 12, the predictive results agreed well with the experimental ones, and the total average relative error was 7.57%, which means that the proposed model can be used for surface roughness prediction. For the relative error of each individual experiment, the maximum value was 73.47% in the second experiment. This could be caused by the micro-breakage of the sharp cutting edges. A new diamond tool was used for the first 12 groups of experiments, and another new tool was used for the last 12 groups of experiments. The cutting edges of the grits were sharp in the initial process. These sharp edges were apt to break with the assistance of vibration, and in this way, numerous micro edges were formed. These micro edges improved the surface quality distinctively, which resulted in an evident decrease of the surface roughness value (from 0.4954 µm in the first experiment to 0.2205 µm in the second experiment). The micro-breakage phenomenon was also mentioned in Ding et al. [24]. Therefore, the abrupt change of the surface roughness value in the second experiment led to a large deviation between the prediction and experimental results. If the second experiment were removed from the verification, the total average relative error could be 4.71%, which indicated a high prediction accuracy of the proposed model.

**Figure 13:**
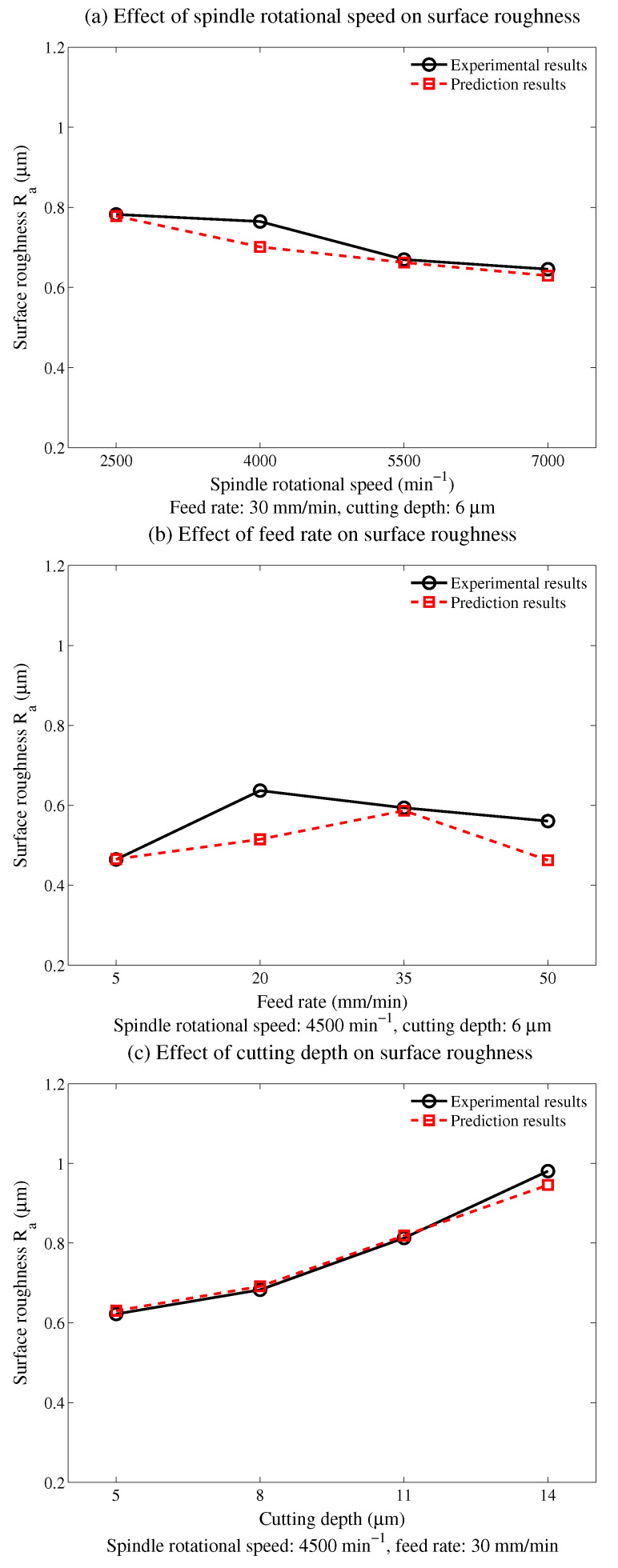
Effects of the input variables on the surface roughness.

The effects of the input variables on the surface roughness are illustrated in Figure 13. From the experimental results, the surface roughness showed a downward trend as the spindle rotational speed increased, while it showed a reverse trend as the cutting depth increased. With increasing feed rate, the surface roughness showed a fluctuating growth trend. Similar effects can also be found in the prediction results, which further validate the proposed model. Similar results were also obtained in previous studies [2,8].

## 5. Conclusions

In this paper, the mathematical expression of the lateral crack propagation path was derived firstly. Afterward, Rayleigh’s probability density function was introduced to describe the penetration depth of the diamond grits. Finally, with the consideration of the ultrasonic vibration characteristics, a comprehensive predictive model for the surface roughness in UVAG of ceramics was proposed. The relationship between input variables and the surface roughness was analyzed both theoretically and experimentally. The following conclusions can be summarized from the study:

1. The prediction results were very consistent with the experimental ones, and the total average relative error was 7.57%. These results verified the validity of the proposed model. Therefore, the proposed model can be applied for surface roughness prediction in the UVAG of ceramics.
2. The effects of the diamond grits’ random distribution, brittle fracture removal, and ultrasonic vibration on the surface roughness were considered during the modeling process. This provided an in-depth understanding of the formation of surface roughness in the UVAG of ceramics and can be also considered as the basis for future parameter optimization.
3. From the developed model, the surface roughness decreased with the rise of the spindle rotational speed, while it showed the opposite trend with increasing cutting depth. Besides, the surface roughness had a fluctuating growth trend with increasing feed rate. Similar results were also obtained in previous studies [2,8].

To further improve the accuracy of the proposed model, reverse engineering methods could be used to obtain the real distribution and shape of the diamond grits. For example, a scanner can be utilized to get the 3D data of the diamond grits, and the distribution characteristics can be derived from statistical analysis. 

## Figures and Tables

**Table 1:** Compositions of the dental zirconia ceramics used for the experiments.

Composition	ZrO${}_{2}$	Y${}_{2}$O${}_{3}$	Al${}_{2}$O${}_{3}$	SiO${}_{2}$	Fe${}_{2}$O${}_{3}$	Na${}_{2}$O
Content (%)	<96	5.30	0.25	≤0.002	≤0.002	≤0.002

**Table 2:** Properties of the dental zirconia ceramics used for the experiments.

Property	Unit	Value
Bending strength	MPa	800–1000
Fracture strength	MPa	1200
Fracture toughness, $K_{IC}$	MPa·m${}^{1/2}$	6
Vickers hardness, $H_{v}$	GPa	12
Young’s modulus, *E*	GPa	210
Density, ρ	g/cm${}^{3}$	6.05

**Table 3:** Details of the experimental design.

Series No.	*n* ($\min^{-1}$)	$v_{f}$ (mm/min)	$a_{p}$ (µm)	Experimental $R_{a}$ (µm)	Predicted $R_{a}$ (µm)
1	2000	30	6	0.4954	0.4941
2	3500	30	6	0.2205	0.3825
3	5000	30	6	0.3223	0.3224
4	6500	30	6	0.3573	0.3228
5	4500	5	6	0.4647	0.4654
6	4500	20	6	0.6368	0.5151
7	4500	35	6	0.5938	0.5867
8	4500	50	6	0.5607	0.4629
9	4500	30	5	0.6221	0.6301
10	4500	30	8	0.6828	0.6913
11	4500	30	11	0.8129	0.8187
12	4500	30	14	0.9806	0.9459
13	2500	30	6	0.7825	0.7782
14	4000	30	6	0.7648	0.7015
15	5500	30	6	0.6698	0.6625
16	7000	30	6	0.6457	0.6296
17	4500	10	6	0.6120	0.6103
18	4500	25	6	0.6509	0.7595
19	4500	40	6	0.4713	0.4710
20	4500	55	6	0.5049	0.4333
21	4500	30	7	0.5404	0.5533
22	4500	30	10	0.5259	0.5483
23	4500	30	13	0.6109	0.6043
24	4500	30	15	0.6829	0.6674

## Abbreviations

UVAG	Ultrasonic vibration-assisted grinding
*A*	Ultrasonic vibration amplitude
$a_{p}$	Cutting depth
*b*	Edge length of a single-diamond grit
$C_{a}$	Diamond grit concentration
$C_{h}$	Crack depth
$C_{l}$	Crack length
*E*	Young’s modulus of the workpiece material
$E(R_{a})$	Expected value of surface roughness
$F_{n}$	Load applied to the abrasive grit
$f_{v}$	Ultrasonic vibration frequency
$H_{v}$	Hardness of the workpiece material
*l*	Evaluation length
*n*	Spindle rotational speed
$N_{a}$	Number of diamond grits
*r*	Position of the grits in the radial direction
*R*	Radius of the tool
$R_{a}$	Surface roughness (arithmetic mean deviation of the assessed profile)
*v*	Poisson’s ratio of the workpiece material
$v_{f}$	Feed rate
*y*	Crack propagation path
α	Angle between two opposite edges of a diamond grit
δ	Penetration depth of a diamond grit into the workpiece
ξ	Geometric factor of the indenter
ρ	Density of the abrasive material
σ	Probability density function parameter
